# A high density GBS map of bread wheat and its application for dissecting complex disease resistance traits

**DOI:** 10.1186/s12864-015-1424-5

**Published:** 2015-03-19

**Authors:** Huihui Li, Prashant Vikram, Ravi Prakash Singh, Andrzej Kilian, Jason Carling, Jie Song, Juan Andres Burgueno-Ferreira, Sridhar Bhavani, Julio Huerta-Espino, Thomas Payne, Deepmala Sehgal, Peter Wenzl, Sukhwinder Singh

**Affiliations:** International Maize and Wheat Improvement Center (CIMMYT), Apdo. Postal 6-641, 06600 Mexico City, DF Mexico; Institute of Crop Science, CIMMYT-China Office, Chinese Academy of Agricultural Sciences, Beijing, 100081 China; Diversity Array Technologies, DArT, Canberra, Australia

**Keywords:** Consensus map, Genotyping-by-sequencing (GBS), QTL mapping, Rust resistance, Segregation distortion, Wheat

## Abstract

**Background:**

Genotyping-by-sequencing (GBS) is a high-throughput genotyping approach that is starting to be used in several crop species, including bread wheat. Anchoring GBS tags on chromosomes is an important step towards utilizing them for wheat genetic improvement. Here we use genetic linkage mapping to construct a consensus map containing 28644 GBS markers.

**Results:**

Three RIL populations, PBW343 × Kingbird, PBW343 × Kenya Swara and PBW343 × Muu, which share a common parent, were used to minimize the impact of potential structural genomic variation on consensus-map quality. The consensus map comprised 3757 unique positions, and the average marker distance was 0.88 cM, obtained by calculating the average distance between two adjacent unique positions. Significant variation of segregation distortion was observed across the three populations. The consensus map was validated by comparing positions of known rust resistance genes, and comparing them to wheat reference genome sequences recently published by the International Wheat Genome Sequencing Consortium, Rye and *Ae. tauschii* genomes. Three well-characterized rust resistance genes (*Sr58*/*Lr46*/*Yr29*, *Sr2*/*Yr30*/*Lr27*, and *Sr57*/*Lr34*/*Yr18*) and 15 published QTLs for wheat rusts were validated with high resolution. Fifty-two per cent of GBS tags on the consensus map were successfully aligned through BLAST to the right chromosomes on the wheat reference genome sequence.

**Conclusion:**

The consensus map should provide a useful basis for analyzing genome-wide variation of complex traits. The identified genes can then be explored as genetic markers to be used in genomic applications in wheat breeding.

**Electronic supplementary material:**

The online version of this article (doi:10.1186/s12864-015-1424-5) contains supplementary material, which is available to authorized users.

## Background

Various marker systems, ranging from low-density restriction fragment length polymorphisms (RFLPs) to high-density single nucleotide polymorphisms (SNPs), have been developed and utilized successfully in wheat for genetic diversity analysis, complex trait dissection, and marker-assisted breeding [1-11]. Anchoring molecular markers on chromosomes and constructing a genetic linkage map are pre-requisites for their utilization in breeding [7,8,10,12-14].

Advances in next-generation technologies have driven the costs of DNA sequencing down to the point that genotyping based on sequence data is now feasible for high diversity, large genome species. Genotyping methods usually involve restriction enzyme digestion of target genomes to reduce the complexity at a reasonable cost [7,15-20]. Davey et al. [17] grouped approaches that apply genome complexity reduction methods into the following classes: reduced-representation sequencing, restriction-site-associated DNA sequencing (RAD-seq), low coverage genotyping including multiplexed shotgun genotyping (MSG) and genotyping by sequencing (GBS). All of these methods are more or less similar technically. The Diversity Arrays Technology (DArT), Canberra, Australia (http://www.diversityarrays.com/), has developed a GBS platform known as DArT-seq, which provides an opportunity to select genome fractions corresponding predominantly to active genes. Restriction enzymes used in this method separate low copy sequences from the repetitive fraction of the genome. These low copy sequences are informative for marker discovery. Representative fragments are then sequenced on Next Generation Sequencing (NGS) platforms [21,22]. Using a combination of restriction enzymes, DArTseq GBS offers affordable genome profiling through generation of high-density SNPs as well as PAV (presence and absence variations) markers [23-26]. In a standard DArT assay, approximately 200,000 genomic fragments are sequenced 10 times, on average, with approximately 2,000,000 tags per sample. As a significant percentage of samples in each experiment are processed in duplicate (to enable stringent marker selection based on scoring reproducibility), all sequence variants that are not legitimate SNP markers are filtered out. Large numbers of additional metadata produced by the analytical pipeline (DArTsoft; DArT P/L, Australia http://www.diversityarrays.com/software.html#dartsoft) make further marker selection and sorting easy, and enable users to choose specific groups of markers that are most useful for their applications [24].

The GBS platform has been used for genetic characterization of more than 40,000 wheat germplasm accessions held by CIMMYT as part of its Seeds of Discovery (SeeD) initiative. A genetic map of GBS markers would be an important prerequisite for trait-based genetic analysis of this large diversity panel. The validity and usefulness of a genetic map depends on its suitability for mapping genomic regions correctly and precisely. In wheat, the Ug99 group of stem rust fungus *Puccinia graminis* Pers. f. sp. *tritici* Eriks. & E. Henn. is described as a major forthcoming threat to global wheat production [9,27]. Mapping and deploying adult plant resistance (APR) genes in popular high yielding but susceptible varieties is needed by wheat breeders. To date, more than 50 APR genes have been identified through QTL analyses [28] and some of them (such as *Sr2*, *Lr46* and *Lr34*) are well characterized and widely used in breeding. Genetic maps that can identify APR genes would be useful for marker-assisted selection (MAS), map-based cloning and detailed molecular characterization in wheat breeding. In this study, three recombinant inbred line (RIL) populations, PBW343 × Kingbird, PBW343 × Kenya Swara and PBW343 × Muu (designated as PB-KB, PB-KS, and PB-MU respectively), were used to anchor the GBS tags to wheat chromosomes. Therefore, the objectives of our investigation were to construct a consensus map of GBS markers and validate known APR genes/QTL against stem rust, yellow rust and leaf rust through mapping, and by comparing them with wheat reference genome sequences recently published by the International Wheat Genome Sequencing Consortium, Rye and *Ae. tauschii* genomes.

## Results

### Marker distribution in the consensus map and three individual maps

Three RIL populations were analyzed using GBS tags. In total, 13123, 18612 and 6936 markers were mapped on PB-KB, PB-KS and PB-MU populations, respectively (Table 1, Additional files 1, 2, 3 and 4). Using genotype data of the three populations, a consensus genetic map was constructed by assigning 28644 markers to wheat chromosomes with 3757 unique positions (Table 1). The maximum ratio of unique positions on the consensus map was located on chromosome 4D (24.4%), whereas chromosome 2D harbored the minimum ratio of unique positions (4.3%). Of the 28644 markers of the consensus map, 32.9%, 56.3% and 10.8% were mapped on the A, B and D genomes, respectively (Table 1). On average, the A, B and D genomes covered distances of 1252.6 cM, 1635.2 cM, and 414.7 cM, respectively.

**Table 1:** **Markers distributed on different chromosomes in three populations and consensus map**

	**Consensus map**				**PBW343/Kingbird**							**PBW343/Kenya Swara**							**PBW343/Muu**						
**Chr.**	**LG** ^**α**^	**Length**	**Total**	**UP** ^**β**^	**LG** ^**α**^	**Length**	**silicoDArT**	**SNP**	**silicoDArT & SNP**	**Total**	**UP** ^**β**^	**LG** ^**α**^	**Length**	**silicoDArT**	**SNP**	**silicoDArT & SNP**	**Total**	**UP** ^**β**^	**LG** ^**α**^	**Length**	**silicoDArT**	**SNP**	**silicoDArT & SNP**	**Total**	**UP** ^**β**^
1A	1	202.57	1275	189	2	152.57	601	102	11	714	128	2	159.47	584	102	14	700	63	2	34.31	128	40	3	171	21
1B	2	233.25	3640	437	1	151.16	2183	106	25	2314	306	2	124.23	2459	86	37	2582	97	1	113.87	1261	134	27	1422	106
1D	1	110.61	274	64	2	60.61	144	20	0	164	39	1	18.95	146	16	1	163	16	1	56.93	57	31	0	88	20
2A	2	160.79	1258	185	1	80.02	393	53	8	454	91	4	157.92	664	60	15	739	77	2	176.28	299	146	13	458	75
2B	1	270.05	3037	403	2	220.05	1480	204	35	1719	253	2	185.33	1605	124	43	1772	107	2	222.92	476	193	20	689	126
2D	2	37.23	1347	58	1	22.29	58	2	0	60	14	1	16.85	1184	34	28	1246	28	2	60.58	52	14	4	70	20
3A	1	255.06	1285	185	1	235.9	443	119	7	569	108	4	191.63	698	116	19	833	75	2	60.58	191	74	11	276	37
3B	2	329.89	2770	411	2	314.89	1146	226	17	1389	262	1	250.53	1487	138	30	1655	121	2	233.94	568	206	27	801	126
3D	1	52.57	433	57	1	52.57	75	10	0	85	24	2	66.38	339	10	3	352	29	1	7.3	9	11	0	20	7
4A	2	152.95	1838	232	1	188	760	105	5	870	155	2	121.16	1215	61	17	1293	90	1	18.25	4	4	0	8	5
4B	1	94.29	595	98	1	94.29	261	59	8	328	61	1	128.25	297	30	7	334	34	1	32.85	38	28	0	66	24
4D	1	42.86	119	29	1	42.86	47	26	2	75	19	1	15.79	42	3	0	45	5	1	18.25	31	7	0	38	10
5A	1	131.56	448	89	1	88.89	91	25	0	116	27	3	83.16	191	36	6	233	34	2	135.77	104	69	5	178	41
5B	1	287.15	1958	276	2	237.15	855	129	20	1004	159	3	210.54	900	79	25	1004	103	2	217.15	294	152	11	457	89
5D	1	31.76	188	19	1	4.86	48	8	1	57	9	1	32.63	127	9	1	137	7	1	13.14	42	18	3	63	6
6A	2	159.17	1521	164	1	132.57	435	47	10	492	72	3	104.21	972	71	14	1057	78	2	128.1	322	117	14	453	51
6B	1	224.42	1989	279	1	224.42	846	85	7	938	145	1	124.26	1227	114	27	1368	115	2	189.42	596	139	12	747	93
6D	1	34.92	316	40	1	25.14	107	13	1	121	17	2	79.13	186	16	4	206	23	1	21.9	35	11	2	48	9
7A	2	190.49	1799	268	1	206.91	718	99	13	830	152	3	220.58	873	94	16	983	79	3	140.15	364	174	13	551	84
7B	2	196.12	2146	217	3	116.66	637	91	13	741	115	2	182.11	1466	98	38	1602	102	1	21.9	185	35	2	222	35
7D	1	104.74	408	57	1	41.14	81	2	0	83	19	2	105.31	279	23	6	308	36	3	62.77	81	27	2	110	26
Total	29	3302.45	28644	3757	28	2692.95	11409	1531	183	13123	2175	43	2578.4	16941	1320	351	18612	1319	35	1966.36	5137	1630	169	6936	1011
A	11	1252.59	9424	1312	8	1084.86	3441	550	54	4045	733	21	1038.1	5197	540	101	5838	496	14	693.44	1412	624	59	2095	314
B	10	1635.17	16135	2121	12	1358.62	7408	900	125	8433	1301	12	1205.3	9441	669	207	10317	679	11	1032.05	3418	887	99	4404	599
D	8	414.69	3085	324	8	249.47	560	81	4	645	141	10	335.04	2303	111	43	2457	144	10	240.87	307	119	11	437	98

LG^α^: Number of linkage groups in each chromosome.

UP^β^: Number of unique positions.

**Figure 1:**
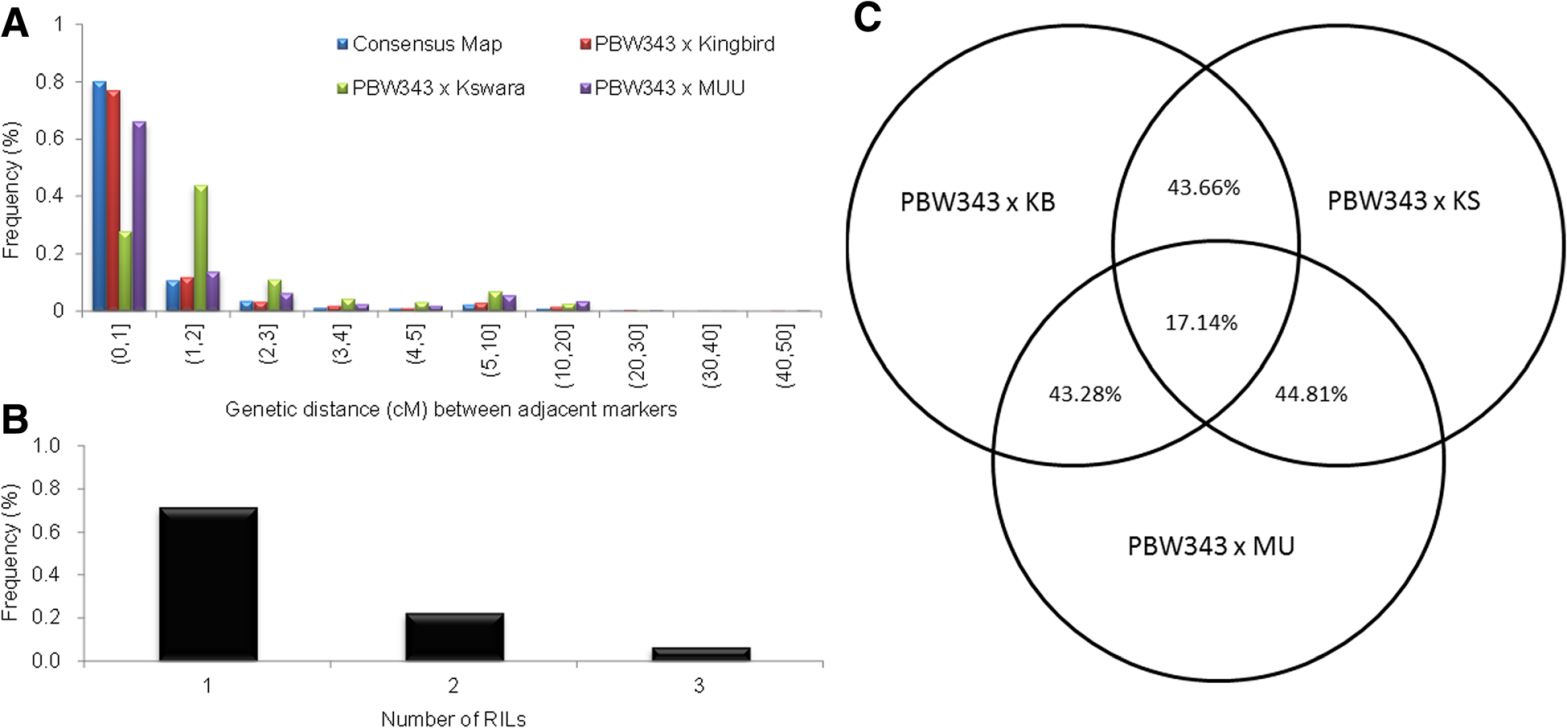
**Frequency of markers on consensus map polymorphic across three populations (A); proportion of common GBS markers across the three RIL populations (B); and frequency of genetics distance (cM) between two adjacent markers on the consensus map (C).** In A, 1 means markers polymorphic in one population, 2 means markers polymorphic in two populations and 3 means markers polymorphic in all three populations.

Total genetic length of the consensus map was 3302.5 cM (Table 1), and average marker distance was 0.88 cM, reached by calculating the average distance between two adjacent unique positions. The length of 3016 marker intervals, corresponding to 80.3% of total marker intervals by 3757 unique positions, ranged from 0 to 1 cM (Figure 1A). In all three populations, 6.4% of markers on the consensus map were found to be polymorphic; 71.4% of markers were polymorphic in one of the three populations, and 22.3% of them were polymorphic in two of the three populations (Figure 1B, and Additional file 5). Compared to the three individual maps, the number of markers in common between any two individual maps was roughly 43% of the number of markers in the map with the least markers of the two (Figure 1C). For example, there were 3002 markers in common between PB-MU and PB-KB, which was 3002/6936 = 43.2% of the number of markers in the PB-MUU map. Also, there were 5729 markers in common between PB-KB and PB-KS, which was 5792/13123 = 43.6% of the number of markers in the PB-KB map. Similarly, there were 1189 markers in common among the three maps, which was 1189/6936 = 17.1% of the number of markers in the PB-MU map.

### Segregation distortion across the three RIL populations

**Figure 2:**
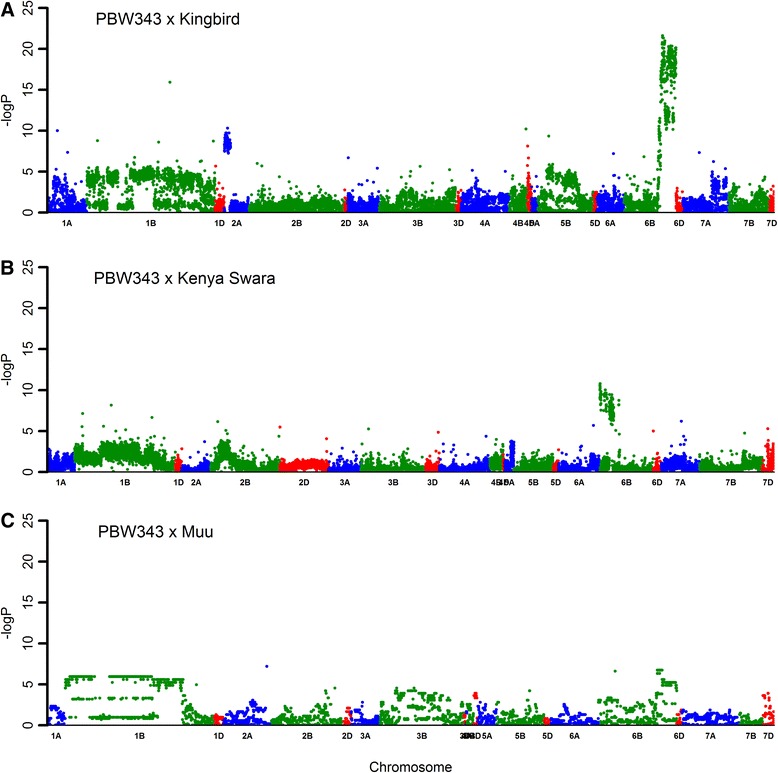
**Manhattan plot of segregation distortion loci mapping across three RIL mapping populations.** **A** is for RIL populations derived by PBW343 and Kingbird; **B** is for RIL populations derived by PBW343 and Kenya Swara; and **C** is for RIL populations derived by PBW343 and Muu.

Segregation distortions were estimated in the three RIL populations. For PB-KB, PB-KS, and PB-MU, 4561 (34.8%), 3686 (19.8%), and 2263 (32.6%) markers, respectively showed evidence of segregation distortion at the 0.05 significance level. The most significant (i.e., -logP) segregation distortion regions (SDRs) were observed in PB-KB, followed by the PB-MU and PB-KS populations (Figure 2). The highest significant SDR was in chromosome 6BL detected in PB-KB, where -logP reached 21.6, and selection favored alleles from PBW343. In both PB-KB and PB-MU, SDRs detected in chromosome 3BS are near the *Sr2* gene, where selection favored alleles from PBW343. An SDR detected in PB-KS on chromosome 7DS was in the region around the *Lr34* gene, designated by Dyck [29], for resistance to leaf rust. In this region, selection favored alleles from Kenya Swara. Among all chromosomes in the three RIL populations, high segregation distortion was observed on chromosome 1B and selection favoring PBW343.

### Distribution of the recombinant inbred lines against wheat rusts

**Figure 3:**
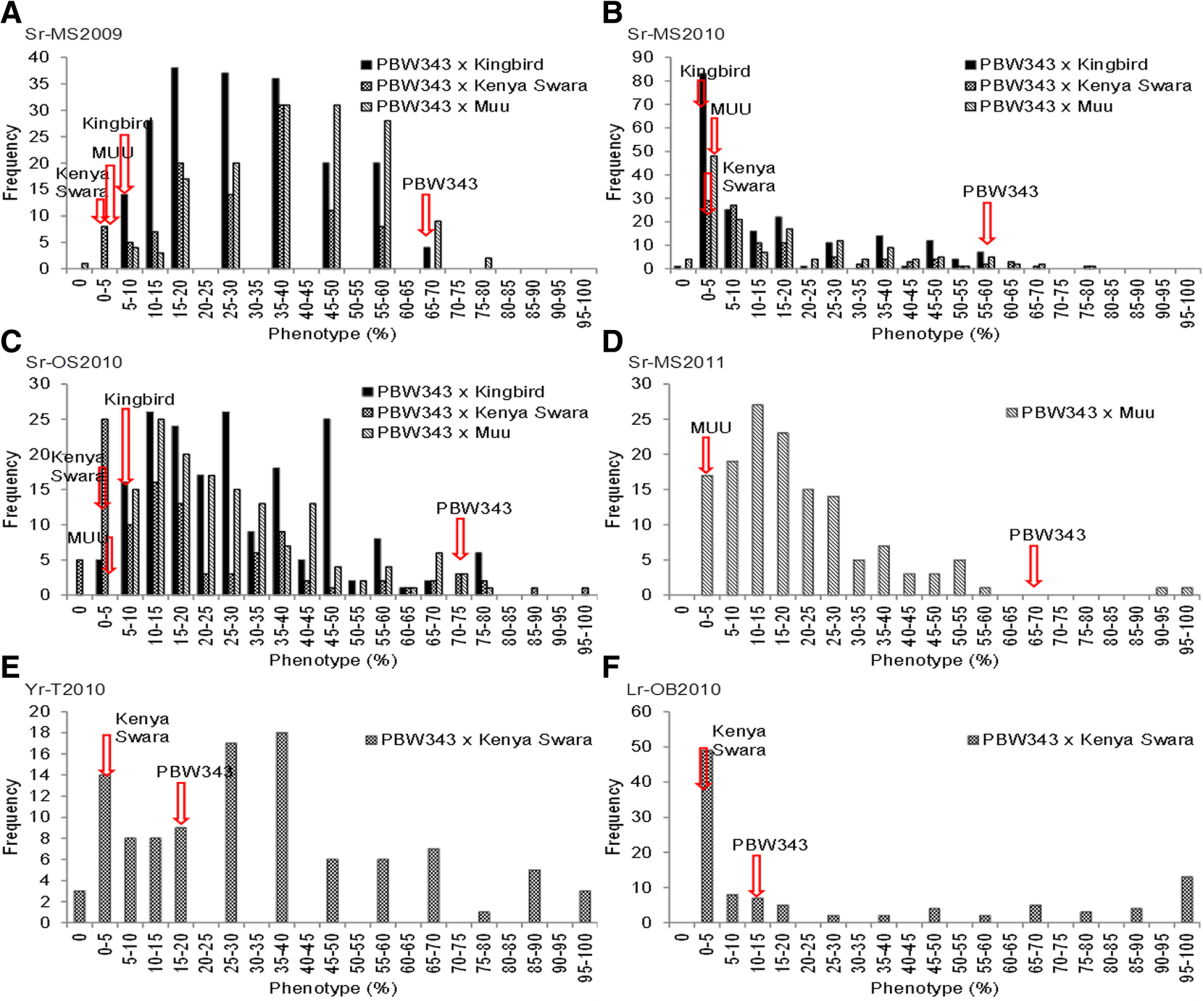
**Phenotypic distribution for rust resistance among population lines in six experiments.** The six experiments were main season 2009 (MS2009), main season 2010 (MS2010), off-season 2010 (OS2010), main season 2011 (MS2011), Toluca, Mexico in 2010 (T2010), and Obregon, Mexico in 2010 (OB2010). Phenotypic values of four parents for each trait in each season are indicated by colored arrows. **A**-**D** are for stem rust (Sr); **E** is for yellow rust (Yr), and **F** is for leaf rust (Lr). Phenotype % represents disease severity.

Large variations for stem rust were observed in the three RILs populations. Screening for yellow and leaf rusts was also carried out (in population PB-KS) to detect pleiotropic loci linked with yellow and leaf rust resistance. Phenotypic variations for leaf and yellow rusts were observed as well, but smaller than those for stem rust (Table 1 and Figure 3). In the MS2009 (main season 2009) screening, percent stem rust severity in the PB-KB, PB-KS and PB-MU populations ranged from 10% to 70%, 1% to 60%, and 0% to 80%, respectively. During MS2010 (main season 2010), it ranged from 0% to 60%, 1% to 80%, and 0 to 80% in the PB-KB, PB-KS and PB-MU populations, respectively; in MS2011 (main season 2011), percent severity varied from 1% to 100% in population PB-MU; and in OS2010 (off season 2010), it was 5% to 80%, 0% to 100%, and 5.6% to 76.3% in PB-KB, PB-KS and PB-MU populations, respectively. Population PB-KS was screened for yellow rust in T2010 (Toluca, Mexico 2010), with percent severity ranging from 0% to 100%, and for leaf rust in OB2010 (Obregon, Mexico 2010), with percent severity ranging from 1 to 100%.

### APR QTLs mapped on individual population linkage maps

**Figure 4:**
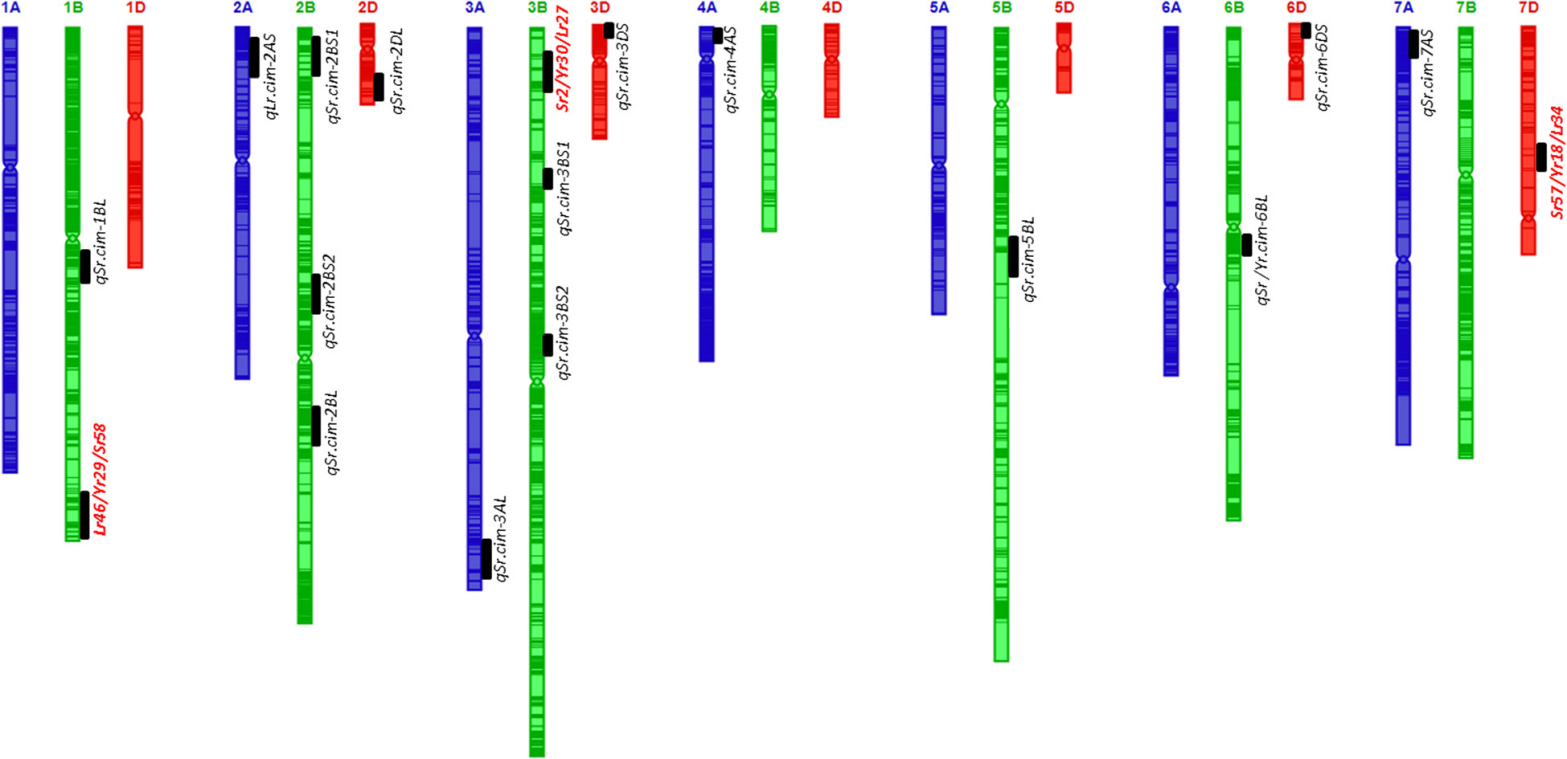
**Identified chromosomal regions harboring APR to stem, yellow and leaf rusts.** QTLs named in red font are well characterized APR genes.

Eighteen QTL regions, projected on the consensus map, were detected to be associated with APR to stem/yellow/leaf rusts in three RIL mapping populations (Table 2 and Figure 4). QTLs were distributed on 13 chromosomes, i.e., 1B, 2A, 2B, 2D, 3A, 3B, 3D, 4A, 5B, 6B, 6D, 7A, and 7D. Well-characterized genomic regions associated with rust resistance (i.e., *Sr58*/*Lr46*/*Yr29*, *Sr2*/*Yr30*/*Lr27* and *Sr57*/*Lr34*/*Yr18*) were identified on chromosomes 1BL, 3BS and 7DS, respectively. Genes *Sr58*, *Sr2* and *Sr57* explained phenotypic variances of up to 15.9%, 37.8%, and 19.5%, respectively (Table 2). Positive alleles for the effects of *Sr2* and *Sr57* were contributed by non-PBW343 parents in all three populations. A positive allele for the effect of *Sr58* from PBW343 was detected in population PB-KS across trials, which may be the reason for this population’s transgressive stem and yellow rust resistance (Table 2 and Figure 4).

**Table 2:** **Mean and range of stem/yellow/leaf rust severity in three RIL mapping populations and their parents over six trials**

**Trial**	**Entry**	**PBW343 x KB**	**PBW343 x KS**	**PBW343 x MU**
Sr-MS2009	PBW343	70	50	60
	non-PBW343	10	1	5
	Ave. (Std.)	32.23 (16.09)	31.20 (15.55)	43.00 (16.63)
	Min-Max	10-70	1-60	0-80
Sr-MS2010	PBW343	60	55	70
	non-PBW343	1	5	5
	Ave. (Std.)	17.25 (17.42)	19.40 (18.35)	20.10 (18.73)
	Min-Max	0-60	1-80	0-80
Sr-OS2010	PBW343	60	75	60
	non-PBW343	10	5	5
	Ave. (Std.)	31.28 (18.02)	22.80 (23.08)	28.40 (17.35)
	Min-Max	5-80	0-100	5.6-76.3
Sr-MS2011	PBW343			70
	non-PBW343			5
	Ave. (Std.)			21.90 (16.39)
	Min-Max			1-100
Yr-T2010	PBW343		15	
	non-PBW343		5	
	Ave. (Std.)		34.60 (26.38)	
	Min-Max		0-100	
Lr-OB2010	PBW343		10	
	non-PBW343		1	
	Ave. (Std.)		29.80 (36.98)	
	Min-Max		1-100	

KS: Kenya Swara.

KB: Kingbird.

MU: Muu.

Apart from these three genic regions, four separately QTLs on chromosome 2 (*QSr.cim-2BS1*, *QSr.cim-2BS2*, *QSr.cim-2BL* and *QSr.cim-2DS*) and chromosome 3 (*QSr.cim-3AL*, *QSr.cim-3BS1*, *QSr.cim-3BS2* and *QSr.cim-3DS*) and single QTL on chromosomes 1 (*QSr.cim-1BL*), 4 (*QSr.cim-4AS*), 5 (*QSr.cim-5BL*), 6 (*QSr.cim-6DL*) and 7 (*QSr.cim-7AS*) were detected (Table 3 and Figure 4). Alleles conferring APR to rust were contributed by PBW343 for *QSr.cim-2DS*, *QSr.cim-3DS*, *QSr.cim-4AS* and *QSr.cim-5BL*, and another nine regions were contributed by non-PBW343 parents. One QTL controlling leaf rust (*QLr.cim-2AL*) was also identified. A pleiotropic locus for APR to stem rust (Ug99) as well as yellow rust was found on chromosome 6BL (*QSr/Yr.cim-6BL*). QTL details in individual populations are presented in Additional files 6, 7, 8, 9 and 10. Genotypic and phenotypic data for QTL mapping in the three RIL populations can be found in Additional files 11, 12 and 13.

**Table 3:** **Additive QTL identified with adult plant resistance to stem/yellow/leaf rust resistance in three RIL mapping populations based on consensus map**

**Population**	**Trial**	**Chr**	**Pos.**	**Marker interval**	**Interval**	**LOD**	**Add**	**PVE (%)**	**QTL/Gene**
PBW343 x MU	Sr-MS2011	1BL	111.5	4260657 - 1110373	1.97	2.61	-3.78	5.34	*QSr.cim-1BL*
PBW343 x MU	Sr-MS2010	1BL	112.5	4260657 - 1110373	1.97	3.27	-4.51	5.78	
PBW343 x KS	Sr-MS2010	1BL	51.6	2289154 - 982224	1.05	3.89	7.37	15.9	*Sr58/Yr29*
PBW343 x KS	Sr-OS2010	1BL	48.3	7348297 - 999754	0.30	3.01	5.93	6.52	
PBW343 x KS	Yr-T2010	1BL	48.3	7348297 - 999754	0.30	2.62	8.14	9.35	
PBW343 x KS	Lr-OB2010	2AL	12.5	4409864 - 1123745	3.04	2.62	-7.37	3.96	*QLr.cim-2AL*
PBW343 x MU	Sr-MS2010	2BS	4.25	4989818 - 1088282	4.73	3.59	-4.83	6.68	*QSr.cim-2BS1*
PBW343 x KB	Sr-MS2010	2BS	120.6	2256042 - 988615	1.56	2.63	-3.51	4.06	*QSr.cim-2BS2*
PBW343 x KS	Sr-MS2009	2BL	165.5	2277655 - 1772231	0.22	2.62	-4.84	9.08	*QSr.cim-2BL*
PBW343 x MU	Sr-MS2009	2DS	28.5	989323 - 1242814	2.19	3.4	5.36	10.31	*QSr.cim-2DS*
PBW343 x KS	Sr-MS2010	3AL	229.0	3941600 - 1252939	2.46	3.12	-6.63	13.16	*QSr.cim-3AL*
PBW343 x KB	Sr-MS2009	3BS	19.5	1057406 - 1280418	6.87	13.1	-7.94	24.45	*Sr2*
PBW343 x KB	Sr-MS2010	3BS	19.5	1057406 - 1280418	6.87	17.1	-10.12	33.95	
PBW343 x KB	Sr-OS2010	3BS	19.5	1057406 - 1280418	6.87	16.3	-10.39	33.39	
PBW343 x KS	Sr-OS2010	3BS	29.05	1106039 - 1314625	13.40	4.95	-8.38	13.3	
PBW343 x MU	Sr-OS2010	3BS	28.89	1106039 - 1140316	4.21	15.7	-10.63	37.79	
PBW343 x MU	Sr-MS2009	3BS	25.8	1280418 - 1035021	1.05	2.84	-4.93	8.72	
PBW343 x MU	Sr-MS2011	3BS	20.3	1321267 - 1280418	6.87	9.3	-7.88	23.24	
PBW343 x KS	Sr-MS2009	3BS	96.6	2296999 - 3022044	1.40	2.79	-4.87	9.66	*QSr.cim-3BS1*
PBW343 x KB	Sr-MS2010	3BS	170.3	1258147 - 993237	1.71	2.69	-3.66	4.42	*QSr.cim-3BS2*
PBW343 x KS	Sr-MS2010	3BS	144.0	3222133 - 4991367	2.82	2.77	-6.49	12.62	
PBW343 x KS	Sr-OS2010	3DS	14.0	2327992 - 1148522	0.98	3.23	6.05	6.89	*QSr.cim-3DS*
PBW343 x KB	Sr-OS2010	4AS	4.0	1092224 - 3945735	3.40	2.81	4.12	5.18	*QSr.cim-4AS*
PBW3433 x KB	Sr-OS2010	5BL	148.0	4410058 - 1161136	1.72	3.85	5.48	9.28	*QSr.cim-5BL*
PBW343 x MU	Sr-OS2010	5BL	156.0	1298718 - 1025982	0.57	3.88	5.14	7.88	
PBW343 x MU	Sr-MS2011	5BL	156.0	1298718 - 1025982	0.57	2.54	4.35	6.3	
PBW343 x KS	Sr-OS2010	6BL	93.1	984306 - 3025087	2.82	2.69	-4.87	5.89	*QSr/Yr.cim-6BL*
PBW343 x KS	Yr-T2010	6BL	95.0	984306 - 3025087	2.82	2.54	-3.66	8.99	
PBW343 x KS	Sr-MS2010	6DL	0.74	1096393 - 1128614	11.69	4.5	-8.53	21.73	*QSr.cim-6DL*
PBW343 x KS	Sr-OS2010	6DL	0.74	1096393 - 1128614	11.69	2.59	-5.49	5.69	
PBW343 x KS	Sr-OS2010	7AS	3.50	4989858 - 989662	4.00	3.91	-6.74	8.52	*QSr.cim-7AS*
PBW343 x MU	Sr-MS2010	7AS	2.36	1240640 - 4990306	2.86	3.6	-4.99	7.13	
PBW343 x KS	Lr-OB2010	7DS	61.0	1128052 - 4991056	3.16	9.53	-17.21	19.5	*Lr34*

KS: Kenya Swara.

KB: Kingbird.

MU: Muu.

Marker intervals: Numbers correspond to the GBS based SNP tags.

Pos.: Position of QTLs on chromosomes in centi-Morgan.

Intervals: Centi-morgan distance between two markers of the interval.

MS: Main season; OS: Off season; Sr: Sem rust; Yr: Yellow rust; Lr: leaf rust.

### Comparison of consensus map with wheat genome reference sequence

**Figure 5:**
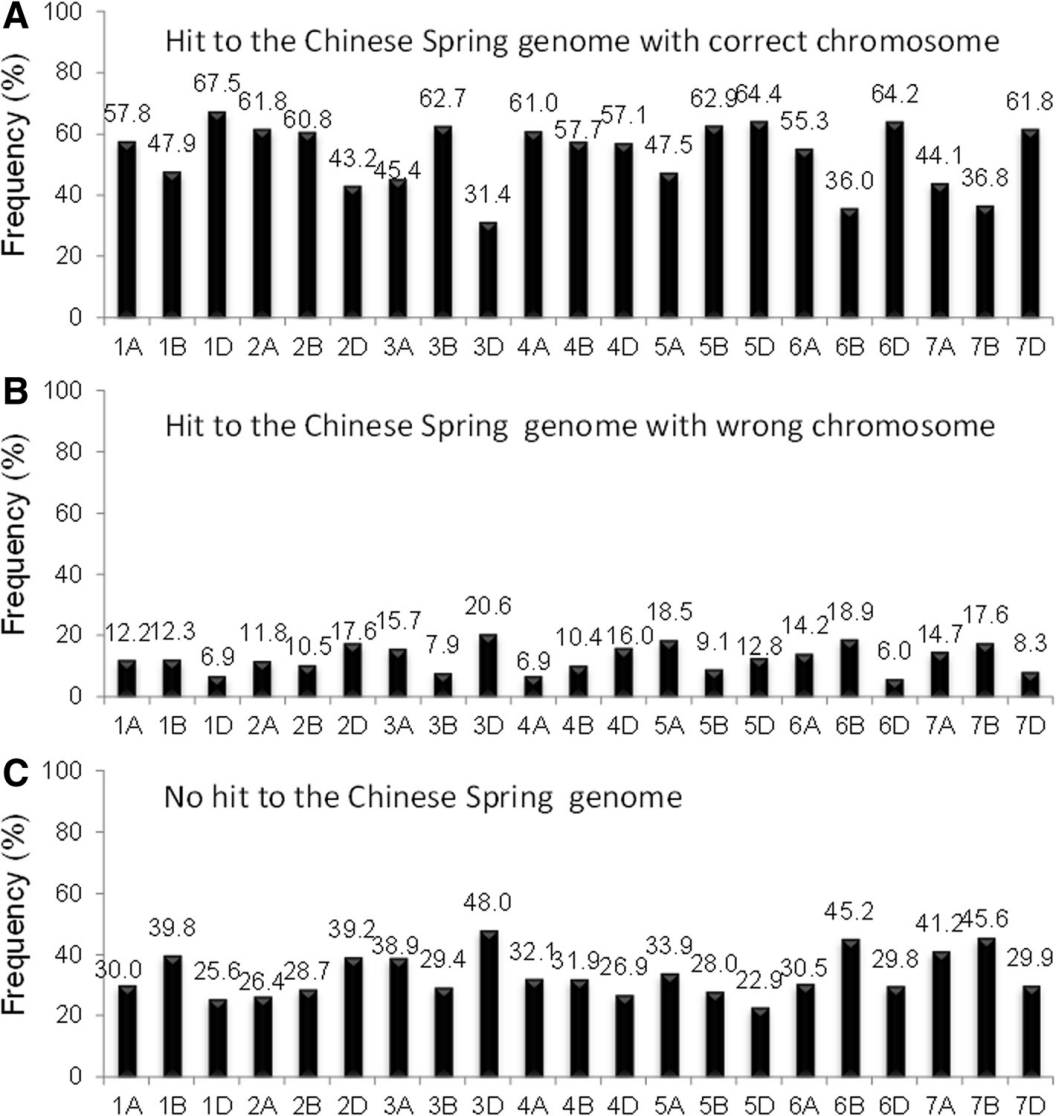
**Frequency of the sequences of GBS markers that were mapped to correct chromosome in the Chinese Spring genome.**

**Figure 6:**
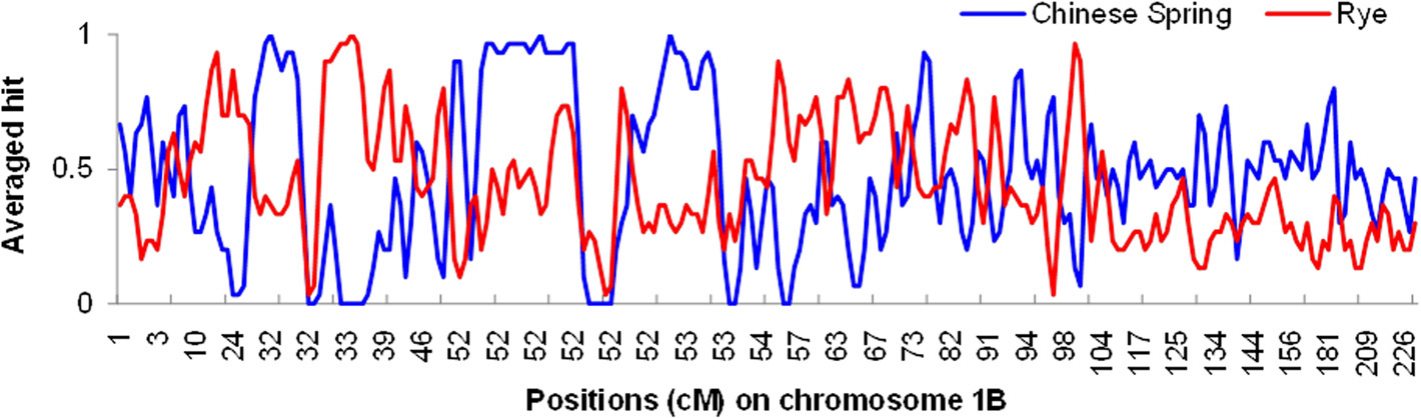
**Averaged hits to the Chinese Spring genome, and rye genome on across chromosome 1B within sliding window with 30 markers in length and 15 markers overlapped between neighboring windows.**

A draft of the wheat genome sequence was published very recently [30]. To verify the consensus map in this study, we BLAST the sequences of 28644 GBS markers (Additional file 14) against the genome sequence of Chinese Spring [30], rye, and the D genome of *Ae. tauschii* with E-value < 1e-5. In general, sequences of 3619 GBS markers (that is, 34.9% of the total GBS markers) cannot hit to the genome sequence of Chinese Spring, and sequences of 12.6% of GBS markers cannot be mapped to the same chromosome in the genome sequence of Chinese Spring as that on the consensus map. Genome-wide sequences of 52.4% of GBS markers can be mapped to the same chromosome in the Chinese Spring genome sequence as that on the consensus map (Figure 5). This ratio ranges from 31.4% to 67.5% across the 21 wheat chromosomes (Figure 5). For chromosome 1B, there was a very clear enrichment for rye hits indicating the 1B/1R translocation (Figure 6). There were increased hits to *Ae. tauschii* on the D genome (Additional file 15).

## Discussion

GBS is a preferred high-throughput genotyping method involving targeted complexity reduction and multiplex sequencing to produce high-quality polymorphism data at a relatively low cost per sample. Three RIL populations sharing one common parent (PBW343) were genotyped using the GBS approach. A consensus genetic linkage map distributed by 28644 markers was developed with 3757 unique positions (13.1% of the total number of markers) covering a 3302.45 cM genetic distance (Table 1; Additional files 1, 2, 3,4 and 5). Recently, a wheat genetic map of 40,267 SNP markers was reported [10] where data were generated with the help of SNP iSelect array comprising ~ 90,000 SNPs. On this map, 13.7% of SNPs were specified to unique positions. The percentage of unique positions in the two maps was comparable. Fewer numbers of unique markers on chromosomes were partially due to the lack of polymorphic markers evenly distributed on wheat genome, and the lack of recombination events captured by these populations.

Compared with the A and D genomes, in the B genome, the maximum percentage of the total number of markers (56.3%; Table 1), the maximum percentage of the total number of unique positions (56.5%; Table 1), the longest genetic length (1635.2 cM), and the maximum number of detected QTL regions can be observed (10 out of 18; Table 2 and Figure 4). These results indicate that most of the recombination events happened on the B genome, which was in accordance with previously reported genetic maps [8,10,23,28,31] and also with genome size, since the B genome is the largest, followed by genomes A and D. Variation in the D genome of bread wheat is consistently low [9-11]. In the present study, 10.8% of markers on the consensus map were located on the D genome (Table 1). Yet in some regions (chromosome 3D in the PB-KB and PB-KS populations, and chromosome 7D in three populations, etc.), a high number of markers with unique positions can be observed (Additional files 1, 2, 3 and 4). Four QTL regions were detected on the D genome (Table 2). This map can be a useful resource for finding more genes located on the D genome to dissect the traits of interest.

In terms of the marker distribution across populations, the highest number of polymorphic markers was available in the PB-KS population, followed by PB-KB and PB-MU (Table 1). When comparing the maps from different populations, the number of markers in common between any two maps was approximately 43% of the number of markers on the smaller map (Figure 1C). The number of markers on all three maps was 17.1% of the number of markers on the smallest map. The reduced percentage of markers in common to all maps may be due to the known structural diversity among the parents and the varying recombination frequency patterns across the genome for the three crosses. These trends indicate PBW343 may have the largest genetic distance from Kenya Swara, compared with Kingbird and Muu.

Although we had 28644 markers on the consensus map, polymorphism markers are still lacking in some chromosome regions of the respective RIL population. Possible reasons are: (1) vast parts of the chromosomes of the *Triticeae* are recombination deserts (the so-called genetic centromeres) [30], so most meiotic recombination events occur in genomic regions that correspond to ~20% of the chromosome length, while there is little recombination in 1/3 to 2/3 of the chromosome; in the region of recombination deserts, it is difficult to explore polymorphic markers; and (2) the different variations among four parental genomes, and different genetic distances between PBW343 and the other three parents (Table 1 and Figure 2). In our study, the numbers of linkage groups for the consensus map and each of the three mapping populations were 29, 28, 43, and 35, respectively (Table 1). If two markers are physically located on the same chromosome but very far away from each other, it is very likely that they will act as unlinked loci in a population of limited size. Due to the recombination desert between them, they will fall into different linkage groups.

A popular variety in South Asia, PBW343 is known for harboring the 1B/1R translocation. On the consensus map, there are 3640 markers located on chromosome 1B, with around 2251 markers on its short arm (1BS) and 1389 markers on its long arm (1BL). Only 12.0% of 3640 markers on chromosome 1B were located uniquely, which is a low proportion across 21 chromosomes. Looking at chromosome 1B specifically, 9.6% of markers are located uniquely on chromosome 1BS, while 16.3% of markers are located uniquely on chromosome 1BL. In other words, the vast majority of markers on chromosome 1BS are represented in the map as clusters of co-segregating markers. Interestingly, high segregation distortion was observed in the three populations on chromosome 1B as compared to the others (Figure 2). Also, regions on chromosome 1B having low hits to the Chinese Spring genome clearly had high hits to the rye genome (Figure 6). These phenomena could be assigned to the 1B/1R rye translocation in PBW343. In addition to the 1B/1R translocation, translocations 2D/2R and 7B/4R have also been reported in wheat. Rye has been used extensively in CIMMYT wheat breeding, and the three parents (PBW343, MUU and Kingbird) used in this study were derived from CIMMYT germplasm. In our study, we could not find evidence of these parents carrying the 2D/2R and 7B/4R translocations.

The consensus map constructed in this study can be used to locate major genes controlling target traits. Phenotypic variation in the three RIL populations suggests polygenic inheritance for APR to stem rust race Ug99 (Table 1 and Figure 3). QTL analysis revealed a couple of APR QTLs against stem rust fungus Ug99, which were mapped as reported. To anchor and determine the relationship between the APR QTLs found in the present study and those found in previous reports (Table 1), we calculated the correlation coefficient of the genotypes of array-based DArT markers and DArT-seq markers on the consensus map. Array-based DArT markers wPt-744022 and wPt-5896 are reported to be linked with APR to stem rust [9]. QTL *QSr.cim-2BS1* reported in this study was flanked by DArT-seq markers, i.e., 4989818 and 1088282. The correlation between 4989818 and wPt-744022 was 0.81 in population PB-MU, which is highly significant. DArT-seq markers 1298718 and 1025982 were the two markers flanking *QSr.cim-5BL* (Table 2). The correlation between wPt-5896 and DArT-seq marker 1298718 was 0.91 in population PB-MU, which is highly significant as well (Additional files 1, 2, 3, 4 and 5). A QTL on chromosome 3BS, which is most likely the *Sr2* gene (in Table 2 designated as *Sr2*) was flanked by DArT-seq markers 1106039 and 1140316 in population PB-MUU. According to the consensus map reported by Yu et al. [31], the *Sr2* gene is 9.2 cM apart from the array-based DArT marker wPt-3761. Marker 1140316 was highly correlated with wPt-3761 in population PB-MUU (correlation coefficient: 0.75). A gene for leaf rust resistance on chromosome 7DS was mapped and flanked by DArT-seq markers 1128052 and 4991056 (Table 2). The correlation between 4991056 and wPt-733087 was 0.69, which is highly significant. Array-based DArT marker wPt-733087 was associated with leaf rust resistance and was found to be 9 cM apart from *Lr34* gene-based marker csLV34 in the PBW343 × Diniza population (Singh et al., CIMMYT, unpublished). In addition to marker co-location for the above mentioned QTLs/gene(s), a recently published consensus map for Ug99 stem rust resistance loci in wheat [31] was compared with QTLs mapped in this study. Since this map did not contain GBS markers, exact co-location could not be made. However, based on the location of APR QTLs on chromosome arms for some of the QTLs, i.e., *QSr.cim-2BS1*, *QSr.cim-2BS2*, *QSr.cim-2BL*, *QSr.cim-2DS, QSr.cim-3AL*, *QSr.cim-3DS*, *QSr.cim-1BL1* and *QSr.cim-6DL* could be co-located with the ones projected on the consensus map of Ug99 stem rust resistance [31]. Singh et al. [9] also reported an APR QTL for Ug99 on chromosome 7AS, which is likely the same as the one on that chromosome in the present study (Table 2 and Figure 4). *QSr.cim-3BS1*, *QSr.cim-3BS2*, *QSr.cim-4AS*, and *QSr/Yr.cim-6BL* were new QTL regions associated with stem rust and yellow rust.

The high density GBS consensus map increased the mapping resolution of linkage mapping. Identified genomic regions (i.e., the genetic region of each QTL’s flanking-marker interval in each individual linkage map) for stem rust resistance ranged from 0.1 to 15.8 cM (Additional file 6). The interval size of 14 of the QTLs was < =1 cM and most of them were  < 5 cM (Additional file 6). QTLs in genomic regions of this size are valuable for further understanding the molecular basis and developing perfectly linked markers. Co-location of the APR QTLs/genes in their respective chromosomal regions (Table 2 and Figure 4) and a high proportion of markers BLAST to the correct chromosomes of the genome sequence of Chinese Spring (Figure 5) indicate the validity and utility of the consensus map.

CIMMYT’s Seeds of Discovery project has characterized more than 40,000 wheat gene bank accessions through the DArTseq GBS platform. The high density GBS consensus map reported in this study is an essential prerequisite for analyzing the GBS data of a large diversity panel. It will facilitate the genetic dissection of important quantitative traits either by linkage mapping as we reported in this paper, or by genome-wide association mapping. GBS markers associated with important traits can be utilized by designing primers according to their sequence, for genomics applications in wheat breeding.

## Conclusions

A high density map of 28644 GBS markers using genotypic data of the three RIL populations with a common parent, PBW343. Total genetic length of map was 3302.5 cM with 3757 unique positions, and the average marker distance was 0.88 cM by calculating the averaged distance between two adjacent unique positions. The length of marker intervals ranged from 0 to 28.3 cM. The number of markers in common between any two individual maps was roughly 43% of the number of markers in the map with the least markers of the two. Significant variation of segregation distortion was observed across three populations. Three genes (*Sr58/Lr46/Yr29*, *Sr2/Yr30/Lr27*, and *Sr57/Lr34/Yr18*) and 15 published QTL were validated. The common parent PBW343 harbors the 1B/1R translocation, and there was a very clear enrichment for rye hits on chromosome 1B. Also, there were increased hits to *Ae. Tauschii* D genome. The high-density and better quality of genetic maps will advance the genetic studies of complex trait in wheat and facilitate genomics-assisted breeding.

## Methods

### Plant materials

Three RIL populations were used for consensus map construction and QTL analysis for rust resistance. Moderately susceptible bread wheat (*Triticum aestivum*) parent PBW343 was used as a common parent and crossed with three other bread wheat APR parents: Kingbird, Kenya Swara, and Muu. PBW343, a major variety in India, is a selection (GID2430154) from CIMMYT line Attila with the pedigree Nord Deprez/VG9144//Kalyansona/Bluebird/3/Yaco/4/Veery#5 [9]. Parents Kingbird and Kenya Swara have maintained high levels of APR to stem rust. Kingbird has shown a high level of APR in field tests conducted at Njoro, Kenya, during the last six cropping seasons, including the 2008 main season, which was characterized by very high stem rust pressure. Muu (pedigree: Pfau/Weaver//Kiritati; GID5090613) was found to be susceptible to wheat stem rust at the seedling stage but adult plants showed low disease severity in response to stem rust race Ug99 during multiple years of field trials in Kenya [32].

### DArTseq™ GBS markers

DArTseq is a GBS platform developed by DArT PL, Canberra, Australia. It is a combination of complexity reduction methods developed initially for array-based DArT and sequencing of resulting representations on next-generation sequencing platforms. For sequencing-based DArT genotyping, two complexity reduction methods optimized for several other plant species at DArT PL (i.e., PstI/HpaII and PstI/HhaI) were used to select a subset of PstI-HpaII and PstI-HhaI fragments, respectively [23]. DNA samples were genotyped twice using two different 4-bp cutters on one end of the RE fragments (HpaII and HhaI). Although the general concept of discovering markers through sequencing of genomic representations was presented over two decades ago [33,34], it was only very recently that the cost and throughput of sequencers reached a point where any GBS-type approach can compete effectively with hybridization-based arrays (DArT and/or SNP; [11]).

The DArTsoft marker extraction pipeline produced large numbers of markers in each of the three populations. Markers were filtered on the basis of reproducibility (that is, the percentage of technical replicate pairs scoring identically for a given marker), call rate (that is, the percentage of samples for which a given marker was scored), and the average read depth (that is, the average number of sequence ‘tag’ counts contributing to the genotype calls for a given marker). Approximately 60% of samples from each population were assayed twice to derive reproducibility scores. The minimum threshold value for reproducibility was 95%, with an average value of 98.5% for SNPs and 99% for silicoDArTs. The minimum threshold value for call rate was 85%, with an average value of 99% for SNPs and 95% for silicoDArTs. The minimum threshold value for Average Read Depth for SNPs was 7, with an average of 18; for silicoDArTs, it was 8, with an average of 17.2. Markers with identical genotypes were placed in redundant bins, and markers with unique genotypes (those that did not belong in a redundant bin) were excluded from the mapping process. This provided an additional quality control step for marker selection. The markers were selected to minimize the number of missing calls as per the selection criterion. The only filtering for ‘false homozygote’ calls was the masking of apparent double crossovers after ordering of the linkage groups. Genotyping errors will present themselves as apparent double crossovers in the ordered map data as ‘singletons’ (data points with genotype scores that differ from those of the immediately preceding and following markers) [35]. For SNP calling, the variants were called within the data only (clustering sequences by sequence similarity), and no external reference genome was used. The sequence defined as the ‘Reference’ for each SNP pair was either the most common sequence in the population or the sequence that had been previously recorded from DArT genotyping analyses of wheat.

### High-density linkage map and consensus map construction

Our three individual maps were constructed using DArT PL’s OCD MAPPING program [36], which implements a marker-ordering algorithm combined with a tunable double cross-over (DCO) masking algorithm. Markers were clustered into linkage groups according to the method described by Anderson et al. [37]. Markers with identical genotypes were placed in redundant bins, and the resulting markers/bins within each linkage group were ordered using the traveling salesman path solver program Concorde [38]. Since silicoDArT markers were dominant markers, while SNP markers were co-dominant markers, silicoDArT markers were separated into paternal and maternal phases. A map was produced for each parent by combining the relevant silicoDArT markers with all of the SNP markers. This resulted in two maps that were joined in a single population consensus map using the SNP markers in common to facilitate consensus map construction. Apparent double-crossovers were masked before reordering the linkage groups and calculating recombination fractions, with Kosambi function used to estimate genetic distances.

Construction of consensus maps presents a challenging problem in wheat due to its structural diversity, particularly the chromosomal structure differences between the parents of mapping populations. We applied DArT PL’s OCD MAPPING program [36] to order DArTseq, array-based DArTs, and SSR markers. We then applied DArT PL’s consensus mapping software [24,36].

### Evaluating stem, leaf and yellow rust severity

The parents, highly susceptible bread wheat check variety, Cacuke and the three RIL populations were evaluated for stem rust severity at the Kenya Agricultural Research Institute (KARI) in Njoro during four crop seasons: main season 2009, main and off-season 2010, and main season 2011, denoted as Sr-MS2009, Sr-MS2010, Sr-OS2010, and Sr-MS2011, respectively. All three populations were evaluated for APR to stem rust, and PBW343 x Kenya Swara was screened for yellow and leaf rusts in one season at one location (Additional file 12). The RILs and parents were sown using a completely randomized design with two replicates. Field plots consisted of two 1-m rows spaced 20 cm apart with a 0.5-m pathway. Approximately 60–70 seeds were sown in each plot. The experimental block was surrounded by a spreader row consisting of varieties differentially susceptible to the *Sr24* virulent variant TTKST. Hill plots of spreaders were also planted in the middle of the pathway on one side of each plot to facilitate uniform disease build-up and spread. On at least two occasions just prior to booting, freshly collected urediniospores suspended in distilled water were injected into culms in the spreader plots (1–3 plants/m) using a hypodermic syringe. Disease response in the field was assessed twice, first, when the susceptible check variety Cacuke displayed 50–60% stem rust severity and subsequently at peak disease development, when Cacuke displayed 100% stem rust at the mid-dough stage of plant growth. Percent disease severity was scored using the modified Cobb Scale [39]. The second rating was considered as the phenotype in this study.

Parents and population lines were evaluated for leaf rust reaction in field nurseries operated by CIMMYT in Ciudad Obregon, Sonora, Mexico, in 2010, denoted as Lr-OB2010. Replicated trials with parents and population lines were grown in Obregon in 2010. Leaf rust severity in each plot was visually scored (near anthesis flowering time) using the modified Cobb scale [39]. For yellow rust screening, parents and population lines were evaluated under field conditions in rust nurseries operated by CIMMYT near Toluca, Edo. Mexico, Mexico, in 2010 and in Njoro, Kenya, in 2010 and 2011, which were denoted as Yr-T2010, Yr-K2010, and Yr-K2011, respectively. Two replicated rows of parents and population lines were assessed in each trial. Each row was visually scored around anthesis flowering time for yellow rust severity with the percentage of leaf covered with disease infection calculated as described for leaf rust. Considering the low phenotypic variance of yellow and leaf rust resistance in the PB-KB and PB-MU populations (data not shown), we did not use them to do QTL analysis in this study. Phenotypic distribution and correlation of rust resistance across the three RIL populations are shown in Figure 3 and Additional file 12.

### QTL mapping of the individual RIL populations

Inclusive composite interval mapping (ICIM) [40-42] implemented by QTL IciMapping 3.2 (available at www.isbreeding.net) was used to map additive and epistatic QTLs controlling stem, leaf and yellow rust resistance. In ICIM for additive QTL mapping, marker selection was performed just once using stepwise regression and considering all marker information simultaneously; this is a key step in determining the scanning profile. Phenotypic values were then adjusted by all markers retained in the regression equation, except the two markers flanking the current mapping interval [40-42]. Permutation tests were conducted using stem rust in the three RIL populations to determine the criteria for model selection in the first step of ICIM. For all three RILs, the probability of a marker moving into the model corresponding to the overall type I error α = 0.05 was approximately 10^-5^. The probability of a marker moving out of the model was set at twice the probability of a marker moving into the model. The LOD threshold to declare the existence of a QTL was calculated by permutation tests as well. Permutation tests revealed LOD thresholds of 3.50, 3.53, and 3.51 for PB-KB, PB-KS, and PB-MU, respectively. Considering that thresholds retained from permutation tests are always conservative [43], an LOD threshold of 2.5 was used to report QTLs and determine common QTLs across seasons and populations.

For epistatic QTL mapping, we tested all possible pairs of scanning positions by ICIM [41]. That is to say, we can detect digenic interactions regardless of whether the two interacting QTLs have significant additive effects or not. Due to the large amount of variables in digenic QTL mapping, we used a much stricter probability (10^-6^) of a marker moving into the model. The probability of a marker moving out of the model was set at twice the probability of a marker moving into the model. An empirical LOD threshold of 4.0 was used to declare the existence of epistatic QTLs.

### Common QTL across the three RIL populations

Due to the differences in the three individual linkage maps, it was difficult to directly find common QTLs across the three RIL populations based on the QTL or marker position in each linkage map. Therefore, we projected each QTL’s flanking markers to the consensus map. If the flanking markers of one QTL were 15 cM apart from the flanking markers of another QTL on both sides, the two QTLs were declared as common QTLs.

## Availability of supporting data

All the genotype data used for analysis are presented along-with manuscript through Additional files 11, 12, 13 and 14. Also, these files have been shared through lab archives http://www.labarchives.com/bmc. DOI for the GBS data: “10.6070/H4Z31WNT”; DOI for the GBS tag sequence: “10.6070/H4TD9V9T”.

## Additional files

Additional file 1:
**Chromosome locations and positions of markers in consensus map constructed in the study.**
Additional file 2:
**Chromosome locations and positions of markers in linkage map of PBW343 x Kingbird population.**
Additional file 3:
**Chromosome locations and positions of markers in linkage map of PBW343 x Kenya swara population.**
Additional file 4:
**Chromosome locations and positions of markers in linkage map of PBW343 x Muu population.**
Additional file 5:
**Details of markers in consensus map, and in linkage maps of PBW343 x Kingbird, PBW343 x Kenya swara, and PBW343 x Muu populations.**
Additional file 6:
**Additive QTLs identified for rust resistance in three RIL populations.**
Additional file 7:
**LOD profile from QTL mapping across the three RIL populations for stem rust.**
Additional file 8:
**LOD profile from QTL mapping in PB-KS for yellow rust.**
Additional file 9:
**LOD profile from QTL mapping in PB-KS for leaf rust.**
Additional file 10:
**Measured traits across seasons and populations, and their correlations across trials.**
Additional file 11:
**Genotypic and phenotypic data used for QTL mapping in PBW343 x Kingbird population.**
Additional file 12:
**Genotypic and phenotypic data used for QTL mapping in PBW343 x Kenya Swara population.**
Additional file 13:
**Genotypic and phenotypic data used for QTL mapping in PBW343 x MUU population.**
Additional file 14:
**Sequences of GBS markers on the consensus map.**
Additional file 15:
**Averaged hits to the Chinese Spring genome, rye genome, and D genome of Ae. Tauschii across 21 chromosomes within sliding window with 30 markers in length and 15 markers overlapped between neighboring windows.**

## Abbreviations

APR: Adult plant resistance
DCO: Double cross-over
DArT: Diversity arrays technology
GBS: Genotyping-by-sequencing
ICIM: Inclusive composite interval mapping
MAS: Marker-assisted selection
MSG: Multiplexed shotgun genotyping
NGS: Next generation sequencing
PAV: Presence and absence variations
RAD-seq: restriction-site-associated DNA sequencing
RIL: Recombinant inbred line
RFLPs: Restriction fragment length polymorphisms
SNPs: Single nucleotide polymorphisms
SDRs: Segregation distortion regions
