# Supplementary material for: A high density GBS map of bread wheat and its application for dissecting complex disease resistance traits
Source: BMC Genomics. 2015 Mar 19;16(1):216. doi: 10.1186/s12864-015-1424-5 (PMC4381402; doi:10.1186/s12864-015-1424-5)
Supplement: Additional file 6: — Additive QTLs identified for rust resistance in three RIL populations. [file 12864_2015_1424_MOESM6_ESM.docx]

Table S6. Additive QTL identified for rust resistance in three linkage maps

| Population | Season | Chr. | Position (cM) | Interval Position | Left Marker | Right Marker | LOD | PVE (%) | Add |  |
| --- | --- | --- | --- | --- | --- | --- | --- | --- | --- | --- |
| PBW343 x Kenya Swara | Sr-MS2010 | 1B_2 | 52 | (51.58, 52.63) | 2289154 | 982224 | 3.89 | 15.90 | 7.37 |  |
| PBW343 x Kenya Swara | Sr-OS2010 | 1B_2 | 48.3 | (48.12, 48.42) | 7348297 | 999754 | 3.01 | 6.52 | 5.93 |  |
| PBW343 x MUU | Sr-MS2011 | 1B | 109 | (108.39, 113.87) | 2322126 | 5970544 | 2.68 | 5.42 | -3.85 |  |
| PBW343 x MUU | Sr-MS2011 | 1B | 37 | (35.77, 39.42) | 1203198 | 1110373 | 2.61 | 5.34 | -3.78 |  |
| PBW343 x MUU | Sr-MS2010 | 1B | 38 | (35.77, 39.42) | 1203198 | 1110373 | 3.27 | 5.78 | -4.51 |  |
| PBW343 x Kingbird | Sr-MS2010 | 2B | 122 | (120.57, 124.00) | 2256042 | 1020900 | 2.63 | 4.06 | -3.51 |  |
| PBW343 x Kenya Swara | Sr-MS2009 | 2B_2 | 40 | (40.00, 40.00) | 2277655 | 1772231 | 2.62 | 9.08 | -4.84 |  |
| PBW343 x MUU | Sr-MS2010 | 2B | 2 | (1.46, 2.92) | 4991300 | 1088282 | 3.59 | 6.68 | -4.83 |  |
| PBW343 x MUU | Sr-MS2009 | 2D | 29 | (28.47, 30.66) | 989323 | 1242814 | 3.40 | 10.31 | 5.36 |  |
| PBW343 x Kenya Swara | Sr-MS2010 | 3A_4 | 13 | (12.63, 15.79) | 3941600 | 1252939 | 3.12 | 13.16 | -6.63 |  |
| PBW343 x Kingbird | Sr-MS2010 | 3B | 171 | (170.30, 172.01) | 1258147 | 993237 | 2.69 | 4.42 | -3.66 |  |
| PBW343 x Kenya Swara | Sr-MS2010 | 3B | 78 | (77.89, 78.66) | 3222133 | 991453 | 2.77 | 12.62 | -6.49 |  |
| PBW343 x Kingbird | Sr-MS2009 | 3B | 18.86 | (18.86, 18.86) | 1057406 | 1321267 | 13.11 | 24.45 | -7.94 |  |
| PBW343 x Kingbird | Sr-MS2010 | 3B | 18.86 | (18.86, 18.86) | 1057406 | 1321267 | 17.12 | 33.95 | -10.12 |  |
| PBW343 x Kingbird | Sr-OS2010 | 3B | 18.86 | (18.86, 18.86) | 1057406 | 1321267 | 16.34 | 33.39 | -10.39 |  |
| PBW343 x Kenya Swara | Sr-OS2010 | 3B | 20 | (17.89, 28.42) | 1228615 | 1314625 | 4.95 | 13.30 | -8.38 |  |
| PBW343 x MUU | Sr-OS2010 | 3B | 1 | (0.73, 1.46) | 1157327 | 1140316 | 15.74 | 37.79 | -10.63 |  |
| PBW343 x MUU | Sr-MS2009 | 3B | 1 | (0.73, 1.46) | 1157327 | 1140316 | 2.84 | 8.72 | -4.93 |  |
| PBW343 x MUU | Sr-MS2011 | 3B | 1 | (0.73, 1.46) | 1157327 | 1140316 | 9.30 | 23.24 | -7.88 |  |
| PBW343 x Kenya Swara | Sr-MS2009 | 3B_4 | 58 | (57.89, 60.00) | 2296999 | 3022044 | 2.79 | 9.66 | -4.87 |  |
| PBW343 x Kenya Swara | Sr-OS2010 | 3D | 8.49 | (8.49, 8.49) | 1114994 | 1236429 | 3.23 | 6.89 | 6.05 |  |
| PBW343 x Kingbird | Sr-OS2010 | 4A | 1.5 | (1.22, 1.71) | 4990541 | 1092224 | 2.81 | 5.18 | 4.12 |  |
| PBW343 x Kingbird | Sr-OS2010 | 5B | 148 | (147.14, 148.86) | 4410058 | 1161136 | 3.85 | 9.28 | 5.48 |  |
| PBW343 x MUU | Sr-OS2010 | 5B_2 | 47.72 | (47.72, 47.72) | 1298718 | 1025982 | 3.88 | 7.88 | 5.14 |  |
| PBW343 x MUU | Sr-MS2011 | 5B_2 | 47.72 | (47.72, 47.72) | 1298718 | 1025982 | 2.54 | 6.30 | 4.35 |  |
| PBW343 x Kingbird | Sr-MS2010 | 6A | 57 | (25.14, 28.57) | 1865416 | 1092868 | 3.69 | 6.13 | 4.33 |  |
| PBW343 x Kenya Swara | Sr-MS2010 | 6A | 28 | (27.37, 29.47) | 3936023 | 1282909 | 4.01 | 16.89 | -7.51 |  |
| PBW343 x Kenya Swara | Sr-OS2010 | 6B | 99 | (98.41, 100.04) | 3025087 | 1115157 | 2.69 | 5.89 | -5.58 |  |
| PBW343 x Kenya Swara | Sr-MS2010 | 6D_2 | 26 | (11.58, 27.37) | 1096393 | 1128614 | 4.50 | 21.73 | -8.53 |  |
| PBW343 x Kenya Swara | Sr-OS2010 | 6D_2 | 27 | (11.58, 27.37) | 1096393 | 1128614 | 2.59 | 5.69 | -5.49 |  |
| PBW343 x Kenya Swara | Sr-OS2010 | 7A_3 | 85 | (85.26, 86.32) | 1861523 | 5582619 | 3.91 | 8.52 | -6.74 |  |
| PBW343 x MUU | Sr-MS2010 | 7A_3 | 40 | (40.88, 41.50) | 4993162 | 1114040 | 3.60 | 7.13 | -4.99 |  |
| PBW343 x Kenya Swara | Yr-T2010 | 1B_2 | 49 | (48.42, 51.58) | 999754 | 2289154 | 2.62 | 9.35 | 8.14 |  |
| PBW343 x Kenya Swara | Yr-T2010 | 6B | 111 | (110.57, 112.68) | 4990983 | 984306 | 2.54 | 8.99 | -7.91 |  |
| PBW343 x Kenya Swara | Lr-OB2010 | 2A_3 | 38 | (36.84, 40.00) | 1123745 | 1070007 | 2.62 | 3.96 | -7.37 |  |
| PBW343 x Kenya Swara | Lr-OB2010 | 7D_2 | 5 | (3.16, 9.47) | 4991056 | 1125802 | 9.53 | 19.50 | -17.21 | |
