# Supplementary figures and images for: A high density GBS map of bread wheat and its application for dissecting complex disease resistance traits
Source: BMC Genomics. 2015 Mar 19;16(1):216. doi: 10.1186/s12864-015-1424-5 (PMC4381402; doi:10.1186/s12864-015-1424-5)

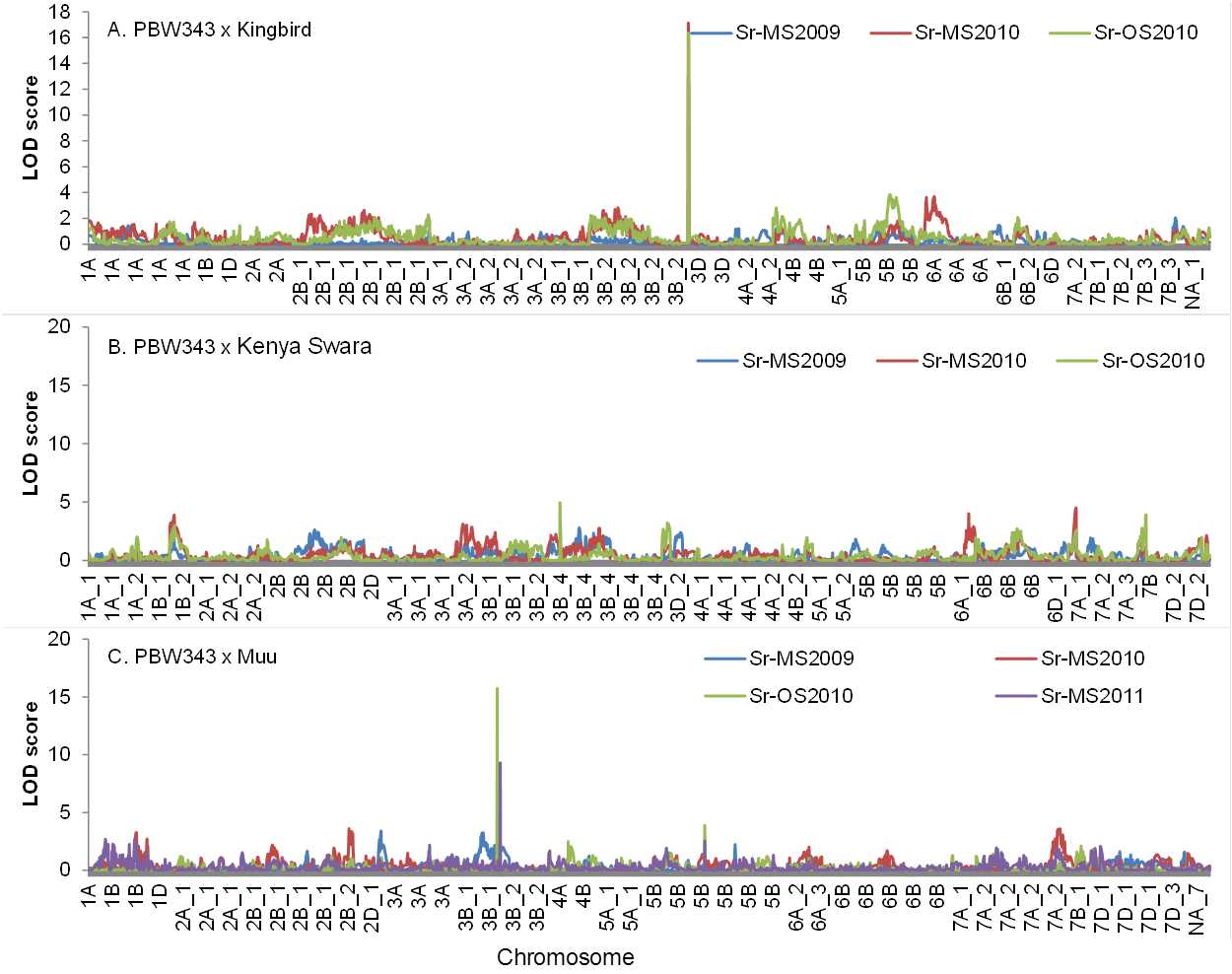

Supplement: Additional file 7: — LOD profile from QTL mapping across the three RIL populations for stem rust. [file 12864_2015_1424_MOESM7_ESM.tiff]

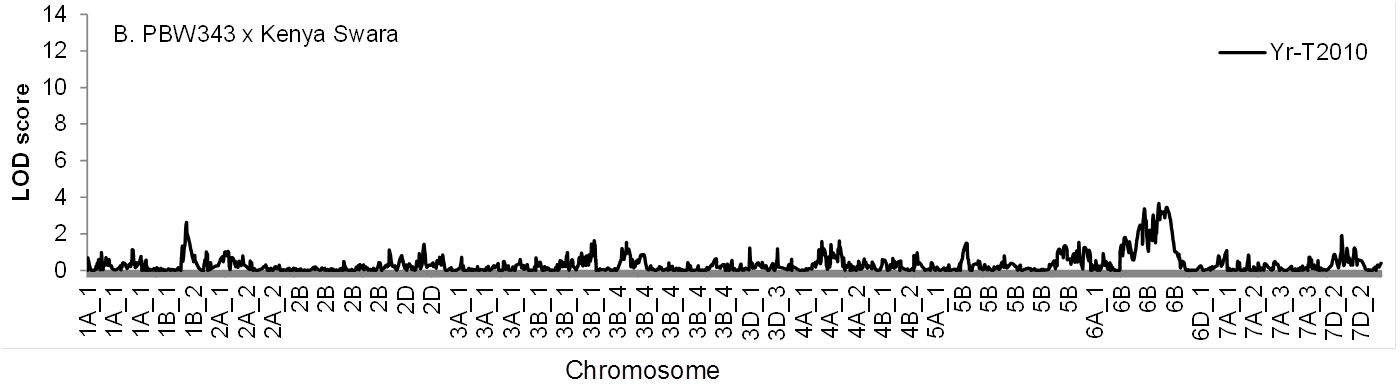

Supplement: Additional file 8: — LOD profile from QTL mapping in PB-KS for yellow rust. [file 12864_2015_1424_MOESM8_ESM.tiff]

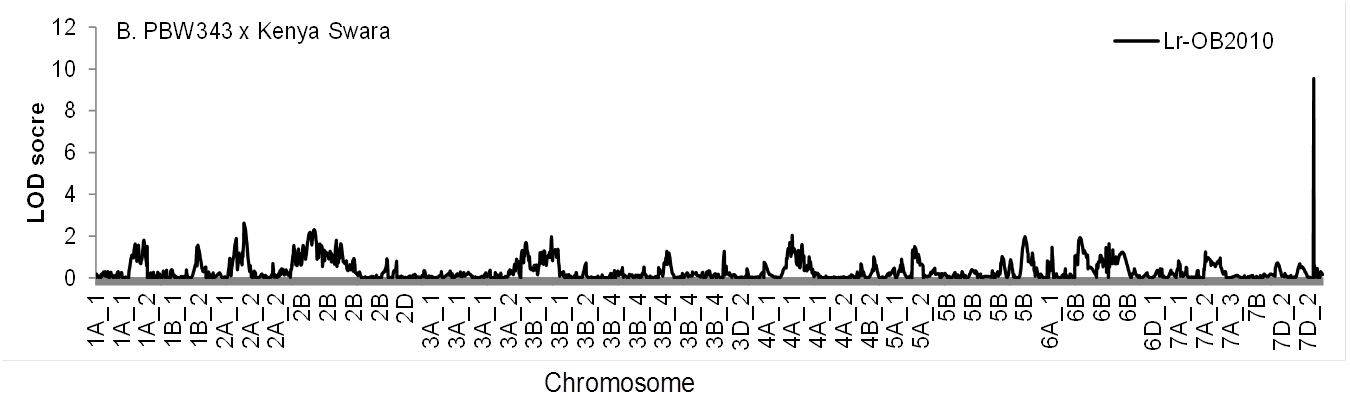

Supplement: Additional file 9: — LOD profile from QTL mapping in PB-KS for leaf rust. [file 12864_2015_1424_MOESM9_ESM.tiff]

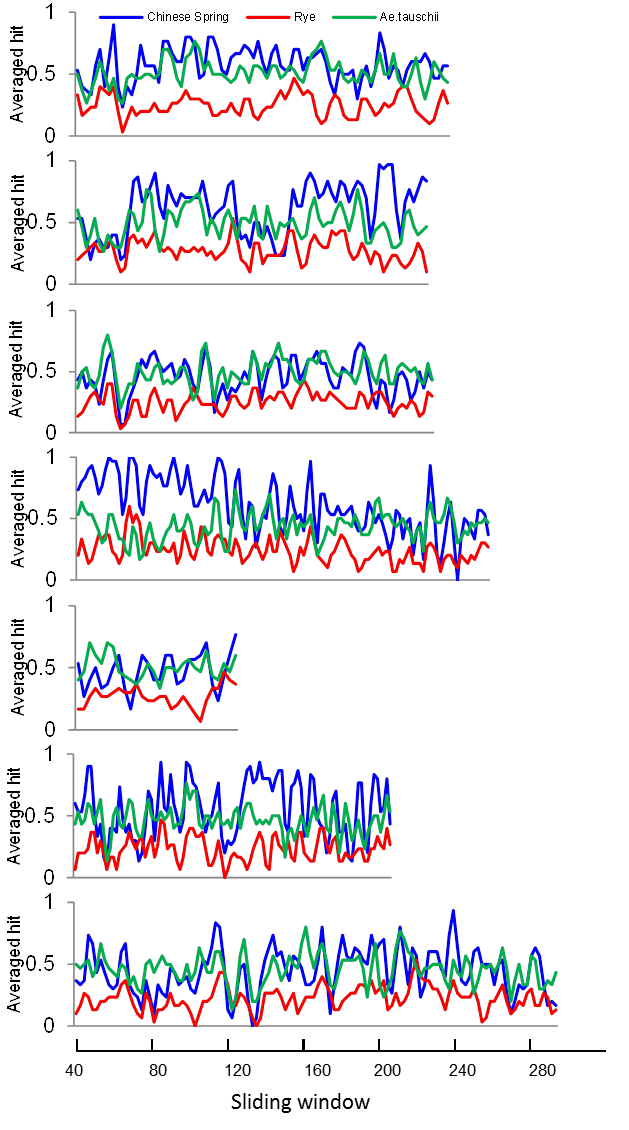

Supplement: Additional file 15: — Averaged hits to the Chinese Spring genome, rye genome, and D genome of Ae. Tauschii across 21 chromosomes within sliding window with 30 markers in length and 15 markers overlapped between neighboring windows. [file 12864_2015_1424_MOESM15_ESM.tiff]
