# Supplementary material for: A high density GBS map of bread wheat and its application for dissecting complex disease resistance traits
Source: BMC Genomics. 2015 Mar 19;16(1):216. doi: 10.1186/s12864-015-1424-5 (PMC4381402; doi:10.1186/s12864-015-1424-5)
Supplement: Additional file 10: — Measured traits across seasons and populations, and their correlations across trials. [file 12864_2015_1424_MOESM10_ESM.docx]

Table S7. Traits measurement across seasons and populations

|  | PBW343 × Kingbird | PBW343 × Kenya Swara | PBW343 × Muu |
| --- | --- | --- | --- |
| Sr-MS2009 | √ | √ | √ |
| Sr-MS2010 | √ | √ | √ |
| Sr-OS2010 | √ | √ | √ |
| Sr-MS2011 |  |  | √ |
| Yr-T2010 |  | √ |  |
| Lr-OB2010 |  | √ |  |

Table S8. Correlations of stem rust (Sr) between different trials for three RIL populations.

| Population | Trial | Sr-2009 | Sr-MS2010 | Sr-OS2010 | Sr-MS2011 |
| --- | --- | --- | --- | --- | --- |
| PB-KB | Sr-2009 | 1 |  |  |  |
|  | Sr-MS2010 | 0.6 | 1 |  |  |
|  | Sr-OS2010 | 0.55 | 0.67 | 1 |  |
|  | Sr-MS2011 |  |  |  |  |
| PB-KS | Sr-2009 | 1 |  |  |  |
|  | Sr-MS2010 | 0.8 | 1 |  |  |
|  | Sr-OS2010 | 0.7 | 0.77 | 1 |  |
|  | Sr-MS2011 |  |  |  |  |
| PB-MU | Sr-2009 | 1 |  |  |  |
|  | Sr-MS2010 | 0.39 | 1 |  |  |
|  | Sr-OS2010 | 0.22 | 0.71 | 1 |  |
|  | Sr-MS2011 | 0.45 | 0.71 | 0.68 | 1 |
